# Mendelian randomization eradicates the causal relationship between educational attainment, household income, and oropharyngeal cancer

**DOI:** 10.3389/fonc.2023.930940

**Published:** 2023-03-02

**Authors:** Li Qi, Wenzhao Bao, Sai Wang, Xiaoxu Ding, Wei Li

**Affiliations:** ^1^ Department of Otorhinolaryngology, The Affiliated Hospital of Inner Mongolia University for the Nationalities, Tongliao, China; ^2^ Department of Otorhinolaryngology, The First Hospital of China Medical University, Shenyang, China; ^3^ Department of Anesthesiology, The Affiliated Hospital of Inner Mongolia University for the Nationalities, Tongliao, China

**Keywords:** oropharyngeal cancer, education attainment, household income, Mendelian randomization, risk factors

## Abstract

**Background:**

It was reported that educational attainment and household income are associated with oropharyngeal cancer. However, whether such an association is causal is still unknown.

**Methods:**

The Mendelian randomization (MR) design was performed to disentangle their causal relationship. Initially, genetic variants proxied for educational attainment and household income were extracted from the largest genome-wide association studies (GWAS), and two oropharyngeal GWAS datasets were used in the discovery and validation stages separately. A reverse MR analysis was carried out to judge whether oropharyngeal cancer affects educational attainment and household income. The results from the two stages were combined using meta-analysis. The heterogeneity and horizontal pleiotropy were appraised using several methods.

**Results:**

All selected genetic variants were valid. In the discovery stage, genetically elevated years of education might decrease the risk of oropharyngeal cancer (IVW OR = 0.148 [0.025, 0.872], p-value = 0.035), while such a result became insignificant in the validation stage (IVW p-value >0.05). Household income cannot change the risk of oropharyngeal cancer at both stages. The reverse MR suggested that oropharyngeal cancer should slightly alter household income (IVW OR = 1.001 [1.000, 1.003], p-value = 0.036) in the discovery set, but the result cannot be replicated in the validation stage. The meta-analysis did not find any significant results either. The results were also assessed by sensitivity analyses, and there was no heterogeneity or horizontal pleiotropy in the analyses. The statistical powers were all above 80% at the discovery stage.

**Conclusions:**

There should be no causal association between educational attainment, household income, and oropharyngeal cancer.

## Introduction

Head and neck cancer (HNC) was the seventh most common cancer worldwide in 2018 and squamous-cell carcinomas of it can be divided into four categories based on the anatomical sites: the oral cavity, sinonasal cavity, pharynx, and larynx (1). Therein, the prevalence of HPV-associated oropharyngeal cancer has been increasing (1). Thus, it is of great importance to prevent oropharyngeal cancer. Several risk factors have been well established for it, such as tobacco consumption and its associated behavior, alcohol intake, human papillomavirus (HPV), vitamin D, educational attainment, low- and middle-income countries, sex behavior, and HPV infection (2, 3). The Global Burden of Disease (GBD) study suggested that South Asia had the highest age-standardized incidence rate (ASIR) and that the risk of oropharyngeal cancer is increasing among females, those aged 15 to 49 years, and people from low/middle-income countries (4). Besides, rural residents and people of black ethnicity have a higher incidence rate and a worse prognosis, which might be attributed to socioeconomic barriers such as insurance and income (5, 6). As for educational attainment, a Danish study found that the incidence of oropharyngeal cancer increased in individuals with a short education (7), and a recent cohort study discovered that those with a lower educational level usually had a lower survival rate (8). However, these traditional observational results tend to be biased by undetectable potential confounders and should be explained with caution (9).

Mendelian randomization (MR) is an emerging epidemiological method for causal inference, using genetic variants as instrumental variables (IV), and has made a substantial contribution to identifying or ruling out risk factors for specified diseases (10). Thanks to the rapid development of genome-wide association studies (GWAS), MR studies have become much more flexible in the two-sample setting (11). A recent MR study, including both univariable and multivariable methods, corroborated the hazardous effect of smoking and alcohol consumption on oropharyngeal cancer (12). Another MR ruled out the causal relationship between 25-hydroxyvitamin D and the risk of oropharyngeal cancer (13). Education has been reported to be associated with oropharyngeal cancer (14, 15), together with income (16). Usually, those with a higher education degree and a higher income are less likely to catch oropharyngeal cancer. These results might be confounded by occupations and other factors (2); however, no study answered the question of whether educational attainment and income are causally associated with oropharyngeal cancer. Traditional observational studies can suffer from unadjusted confounders and reverse causation. Our MR analysis used genetic variants to estimate the causal relationship between educational attainment, household income, and oropharyngeal cancer using a causal inference framework, which should be a suitable method to give causal inference when randomized clinical trials (RCTs) are unavailable and impractical (17).

In this study, we aim to use MR to explore whether genetically-determined educational attainment and household income can causally affect the risk of all forms of oropharyngeal cancer, hoping to explain their causal relationships.

## Methods

### Data source

The GWAS summary statistics of educational attainment were defined as years of education, and this GWAS was performed on 126,559 individuals of European ancestry, adjusting for sex and the interaction term between sex and birth year (18). We extracted GWAS summary statistics of income from a study with 332,050 European participants (GCST009523) where the household income was collected using a 5-point scale corresponding to the total household income before tax, 1 being less than £18,000, 2 being £18,000–£29,999, 3 being £30,000–£51,999, 4 being £52,000–£100,000, and 5 being greater than £100,000 with adjustment of 40 genetic principal components, genotyping array, batch, age, and sex (19). The GWAS summary statistics of oropharyngeal cancer were obtained from two sources: one from the open GWAS, whose ID is “ieu-b-96,” and another from the GWAS catalog (GCST90011806). Therein, the former included 4,018 Europeans, with 1,090 cases and 2,928 controls, and this study adjusted for age, sex, and principal components (20). The latter is a GWAS meta-analysis of the UK Biobank (UKB) and Kaiser Permanente Genetic Epidemiology Research on Adult Health and Aging (GERA) cohorts, consisting of 1,223 European cases and 410,350 European controls, and included age, sex, the first ten genetic principal components, the genotyping array (UKB only), and the reagent kit used for genotyping (Axiom v1 or v2; GERA only) (21). All involved participants were Europeans, sharing the same genetic background, and the causal estimates would not be biased by ancestry-specific heterogeneity. Please note that the information in this study was not collected from specific countries, and all the GWSA data used were summary-level. Details of the GWAS can be found in **Table 1**.

**Table 1:** A summary of genome-wide association studies.

Exposure	Ancestry	Sample size	Covariates	NSNP	R^2^	F	PMID
Educational attainment	European	126,559	sex, sex*birth year	4	0.06	2019.48	23722424
Household income	European	332,050	40 genetic principal components, genotyping array, batch, age, and sex	28	0.07	892.53	31844048
Oropharyngeal cancer (discovery)	European	1,223 European cases and 410,350 European controls	age, sex, first ten genetic principal components, genotyping array	27	0.04	635.1	32887889
Oropharyngeal cancer (validation)	European	1,090 cases and 2,928 controls	age, sex, and principal components	18	0.05	11.69	27749845

NSNP is the number of single nucleotide polymorphisms; R^2^ is the variance of exposure explained by SNPs; F is the F statistic; PMID is the ID of included studies in PubMed.

### Mendelian randomization design

**Figure 1:**
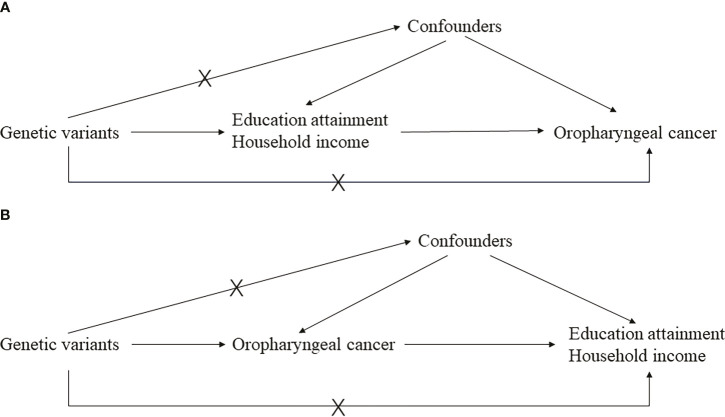
The main design of this study. **(A)** is the basic principal assumptions of the two-sample Mendelian randomization design; **(B)** is the reverse Mendelian randomization design.

Preliminarily, educational attainment and household income were treated as exposures, and oropharyngeal cancer was the outcome. Considering that “GCST90011806” has a larger sample size, we treated it as the discovery set and “ieu-b-96” as the validation set. A reverse MR was performed as well, where oropharyngeal cancer was the exposure and educational attainment and household income were the outcomes, hoping to clarify reverse causation. A meta-analysis was employed to combine the results of both the discovery and validation stages, hoping to obtain a more precise estimation with an enlarged sample size. A brief demonstration of the study design can be found in **Figure 1**.

A single nucleotide polymorphism (SNP) was selected if it reached the genome-wide significance (p-value <5 × 10^−8^) and we further clumped them to obtain independent IVs based on linkage disequilibrium (LD r^2^ = 0.01). An SNP with a low minor allele frequency (MAF <0.01) was removed from further analysis. Mainly, the inverse-variance weighted (IVW) method was utilized to estimate the effect of exposure on the outcome. Besides, MR-Egger regression and weighted-median methods were used as supplementary methods. As heterogeneity and horizontal pleiotropy can usually bias MR estimation, several methods have been applied to control them. Cochran’s Q value was calculated to assess heterogeneity (22). Two methods were utilized to judge horizontal pleiotropy, including the MR-Egger intercept (23) and MR-PRESSO (24). To guarantee that genetic variants explain more variance of exposure than that of the outcome, the MR Steiger test was performed, and we would remove SNPs explaining more variance of outcome (25).

### Statistical analysis and data visualization

The IVW method was used as the main analytical method since it can give the most accurate estimate if all instruments were valid; however, the supplementary methods MR-Egger and weighted-median can give robust estimates in the presence of invalid instruments, and these invalid instruments might introduce heterogeneity and horizontal pleiotropy to the MR estimates if using the IVW method (23, 26). Also, we adopted Cochran’s Q value to assess heterogeneity and used MR-Egger intercept (23) and MR-PRESSO to assess horizontal pleiotropy (24). The main MR statistical analyses were performed using the R package “TwoSampleMR.” The MR-PRESSO analysis was carried out using the R package “MRPRESSO.” The meta-analysis was performed using the R package “meta.” The power for each causal association was calculated using the online Shiny application “mRnd” (27). Data visualization was performed using the R package “forestplot.”

## Results

Generally, most study participants were aged 40–69, and the proportion of females was 54.30% (https://www.ukbiobank.ac.uk/). The number of IVs for educational attainment and household income was 4 and 28, respectively, and the number of IVs for oropharyngeal cancer was 27 and 18, respectively. In this MR study, we did not find a robust causal relationship between household income, years of education, and oropharyngeal cancer. The F statistic of each IV was greater than the empirical threshold of 10, suggesting the results are less likely to be biased by weak instruments. The MR-Steiger test further corroborated the tested causal direction from exposure to the outcome, where the selected genetic variants explained more variance of exposure than that of the outcome.

### Oropharyngeal cancer as the outcome

**Figure 2:**
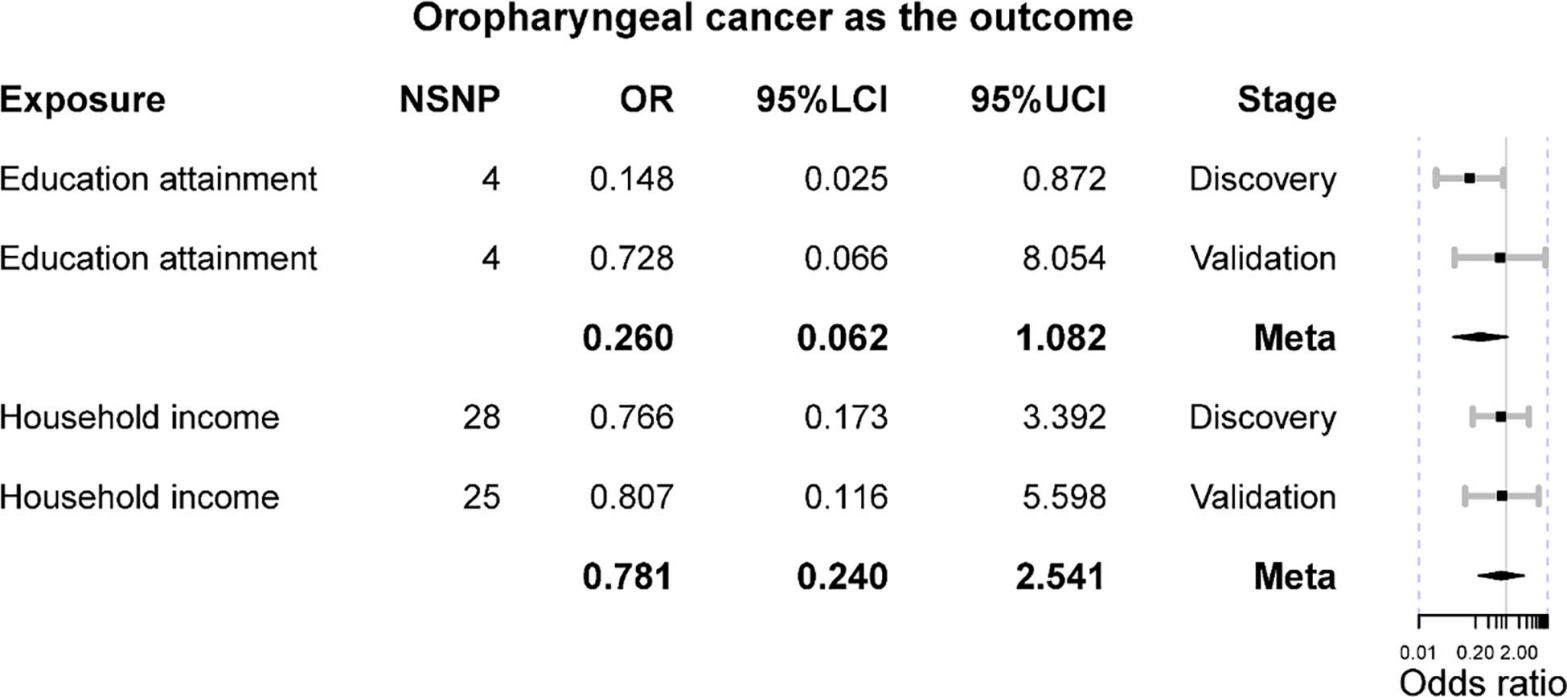
The Mendelian randomization results of educational attainment and household income on oropharyngeal cancer. OR is the odds ratio; 95%LCI is the lower limit of 95% confidence interval; 95%UCI is the lower limit of 95% confidence interval.

The main results indicated that genetically elevated household income cannot affect the risk of oropharyngeal cancer using 28 instruments in the discovery set (IVW OR = 0.766 [0.173, 3.392], p-value = 0.726), and it was confirmed in the validation set with 25 instruments (IVW OR = 0.807 [0.116, 5.598], p-value = 0.828). No significant result was found in the meta-analysis (Meta OR = 0.781 [0.240, 2.541], p-value = 0.681) (**Figure 2**). No heterogeneity was detected in the analysis (IVW Q p-value = 0.183 in the discovery set; Q p-value = 0.769 in the validation set). Furthermore, there were no outliers found by MR-PRESSO, and the MR-Egger intercept was no different from zero, suggesting a paucity of horizontal pleiotropy. Additionally, other analytical methods also confirmed the null association between household income and oropharyngeal cancer, including MR-Egger, weighted median, and weighted mode methods (**Table 2**). The statistical power of these estimates was greater than 80%. Besides, we observed that genetically elevated years of education might decrease the risk of oropharyngeal cancer (IVW OR = 0.148 [0.025, 0.872], p-value = 0.035); however, such results were not replicated in the validation (IVW OR = 0.728 [0.066, 8.054], p-value = 0.796). The meta-analysis also supported a null association (meta OR = 0.260 [0.062, 1.082], p-value = 0.064) (**Figure 2**). Although the results derived from the discovery stage indicated a marginal causal effect where genetically elevated years of education can reduce the risk of oropharyngeal cancer, the complementary methods did not support the IVW result as both MR-Egger and weighted median methods did not implicate statistical significance (p-value >0.05) (**Table 2**). No heterogeneity was detected in both discovery (IVW Q p-value = 0.793) and validation stages (IVW Q p-value = 0.447). Also, there was no heterogeneity in the meta-analysis. Both the MR-PRESSO and MR-Egger intercept tests implicated no horizontal pleiotropy in the causal estimates. The statistical power of these MR estimates for years of education as exposure in the discovery stage was 96%, while it was reduced to 30% in the validation stage.

**Table 2:** The Mendelian randomization results of complementary methods.

			MR-Egger				Weighted median					
Exposure	Outcome	Stage	OR	95%LCI	95%UCI	P	OR	95%LCI	95%UCI	P	P_heterogeneity_	P_pleiotropy_
Household income	Oropharyngeal cancer	Discovery	0.057	0.000	2.613E+03	0.605	1.103	0.158	7.721	0.921	0.183	0.636
Household income	Oropharyngeal cancer	Validation	0.010	0.000	6.155E+03	0.507	0.368	0.022	6.170	0.487	0.769	0.523
Education year	Oropharyngeal cancer	Discovery	3.483E+03	0.000	1.369E+14	0.580	0.105	0.013	0.837	0.033	0.793	0.503
Education year	Oropharyngeal cancer	Validation	0.000	0.000	3.742E+03	0.280	1.229	0.071	21.402	0.888	0.447	0.284
Oropharyngeal cancer	Household income	Discovery	1.002	0.998	1.005	0.386	1.002	1.000	1.004	0.049	0.261	0.943
Oropharyngeal cancer	Household income	Validation	1.003	0.998	1.007	0.239	1.000	0.998	1.002	0.764	0.000	0.118
Oropharyngeal cancer	Education year	Discovery	0.993	0.977	1.009	0.451	0.997	0.984	1.009	0.585	0.853	0.541
Oropharyngeal cancer	Education year	Validation	0.968	0.891	1.051	0.478	0.996	0.982	1.010	0.540	0.836	0.547

OR is the odds ratio; 95%LCI is the lower limit of 95% confidence interval; 95%UCI is the lower limit of 95% confidence interval; P is the p-value of OR; P_heterogeneity_ is the p-value of heterogeneity test; P_pleiotropy_ is the p-value of pleiotropy test.

### Oropharyngeal cancer as the exposure

**Figure 3:**
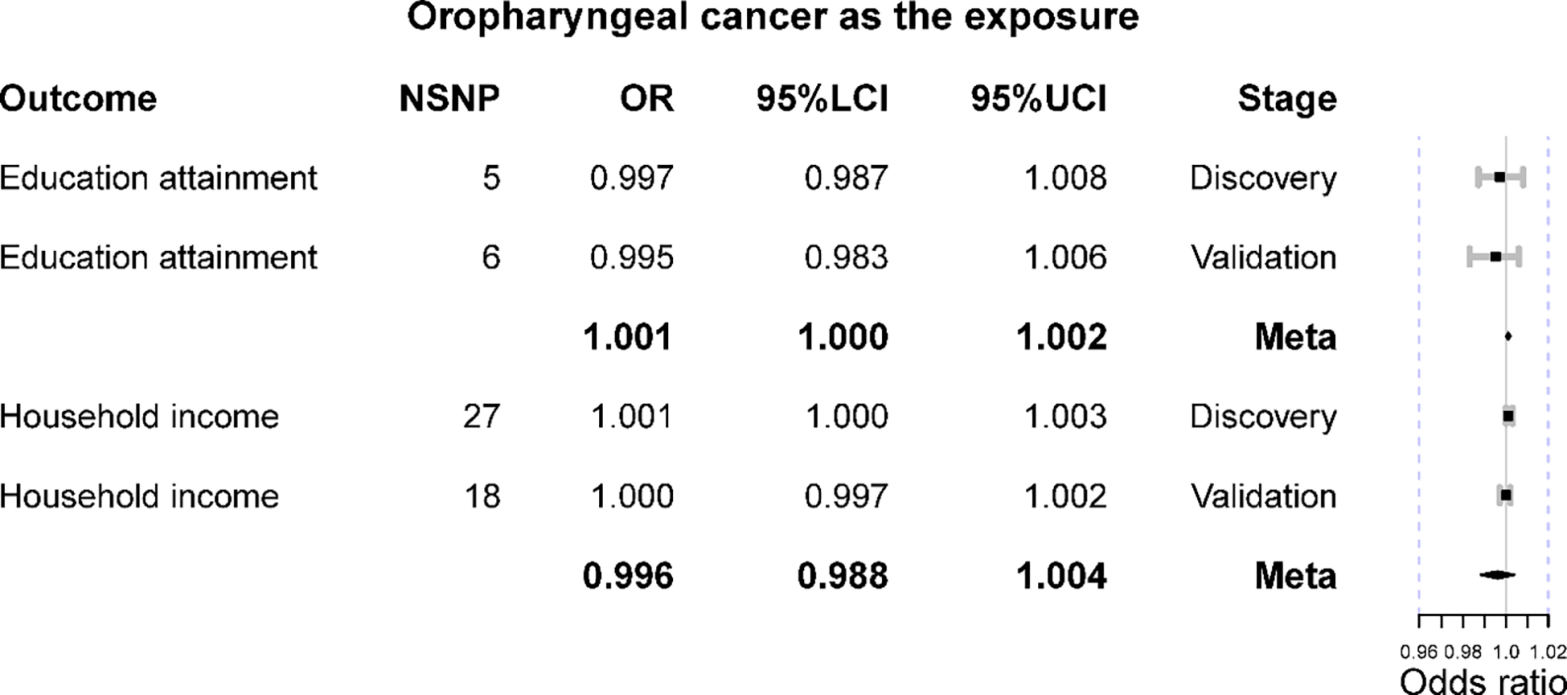
The Mendelian randomization results of oropharyngeal cancer on education attainment and household income. OR is the odds ratio; 95%LCI is the lower limit of 95% confidence interval; 95%UCI is the lower limit of 95% confidence interval.

The reverse MR suggested that genetic predisposition to oropharyngeal cancer should marginally alter household income (IVW OR = 1.001 [1.000, 1.003], p-value = 0.036) in the discovery set; however, it was not corroborated in the validation stage (IVW OR = 1.000 [0.997, 1.002], p-value = 0.732) (**Figure 3**). Furthermore, the meta-analysis was consistent with these results (meta OR = 0.996 [0.988, 1.004], p-value = 0.309). Besides, the result of MR-Egger was not significant (p-value >0.05), while that of the weighted median method was marginally significant (OR = 1.002 [1.000, 1.004], p-value = 0.049) at the discovery stage. MR-PRESSO detected outliers at the validation stage, and the results remained insignificant after removing them. No heterogeneity or horizontal pleiotropy was detected (IVW Q p-value >0.05 and MR-Egger intercept p-value >0.05).

When treating oropharyngeal cancer, its genetic liability cannot alter education year level (IVW OR = 0.997 [0.987, 1.008], p-value = 0.599), and it was corroborated in the replication set (IVW OR = 0.995 [0.983, 1.006], p-value = 0.349). The meta-analysis did not obtain a significant result (meta OR = 1.001 [1.000, 1.002], p-value = 0.108). All the other supplementary methods support such a null association (**Table 2**). No heterogeneity or horizontal pleiotropy was detected (IVW Q p-value >0.05 and MR-Egger intercept p-value >0.05).

## Discussion

Generally, our MR analyses ruled out the causal effect of household income on oropharyngeal cancer. However, higher educational attainment seemed to lower its risk, but the results were not consistent in the two-outcome datasets “ieu-b-96” and “GCST90011806.” The reverse MR analysis indicated that oropharyngeal cancer could not affect income and educational attainment.

In this study, we observed that higher achievement in education might lower the risk of oropharyngeal cancer in the discovery set, which was not replicated in another dataset. Such a discrepancy should be explained by the difference between two oropharyngeal datasets. In this case, the discovery set contained a smaller sample size, which should lead to a null association due to insufficient statistical power. However, further meta-analysis indicated a null association with a pooled large sample size. We postulate that the underlying causal mechanism is likely to be mediated *via* behavioral lifestyle factors and/or psychosocial, physical, and life-course pathways (16). Increased educational attainment has a significant impact on people’s choice of lifetime habits, and such habits have a greater impact on head and neck cancer, such as smoking and alcohol consumption (1). For instance, the frequency and magnitude of smoking and drinking are lower among those with higher educational attainment than among those with lower educational attainment (28, 29). Studies have shown that the carcinogenic substances in cigarettes significantly increase the risk of head and neck cancer, and the risk is synergistically increased when combined with alcohol (30–32). As David et al. reported, 70% of head and neck cancers can be avoided through lifestyle changes, especially by modifying smoking and alcohol consumption behaviors (16). Physical and psychological variables both influence the incidence and prognosis of head and neck cancer significantly (33). Psychological distress is strongly associated with an increased risk of head and neck cancer (34) and has a detrimental effect on the prognosis of individuals with head and neck cancer (35), while physical activity is inversely associated with an increased risk of head and neck cancer (36). Improvements in educational attainment will have a beneficial impact on psychological and physical factors. A Japanese epidemiological study in 2021 showed that increased educational attainment improves people’s psychological health and decreases the incidence of depressive symptoms (37). Additionally, when an individual’s educational level was high, self-reported physical activity had a favorable mediation effect on the association between personal control and health (38). The life course of highly educated individuals is frequently distinct from that of less educated individuals (39). Highly educated individuals have more work opportunities and access to more social resources that help them maintain good health (40, 41), such as improved medical resources for early head and neck cancer screening and earlier lifestyle interventions and medications to reduce their risk of developing head and neck cancer (42).

To our knowledge, no study investigating the effect of head and neck cancer on educational attainment has been published until now. Our study suggested that genetic liability to HNC should not alter a person’s educational attainment. The major explanation should be that educational attainment is closely associated with heritability and nurturing, and diseases should contribute less to it. As previously reported, decreasing educational attainment may relate to heritable syndromes that cause cognitive deficits and impair academic abilities (43). Neurofibromatosis 1, one of the most common hereditary diseases, has been proven to impair cognitive function and can result in lower educational attainment (43). However, HNC is not predominantly caused by genetic factors but rather by acquired characteristics that have a minor influence on the patient’s cognitive ability (2). Hence, HNC has little effect on children’s educational attainment. Apart from affecting children’s education, cancer can also impede adults’ ability to attain higher levels of education. However, the great majority of patients with head and neck cancer already possess advanced degrees (44), which have a negligible effect on the educational attainment of adults. This is also consistent with our finding that HNC does not affect educational attainment.

Socioeconomic risk associations are comparable in magnitude to those of behavioral risk variables across all head and neck malignancies, with the greatest burden of head and neck cancer reported among individuals with the lowest incomes (16). According to the most recent occupational socioeconomic risk association study on head and neck cancer in Europe and South America, occupational socioeconomic status, position, and physical labor are all connected with head and neck cancer (32). However, this discrepancy is due to comparing data from different regions, and different confounding factors exist in different parts of the population, resulting in different results (45). In this paper, the results of the analysis of the corresponding data obtained from the European database found no significant association between income and the incidence of HNC. This may be because Europe’s medical infrastructure is well developed, and people’s habits and lifestyles are different from those of people in developing countries. This may result in affluence not affecting the incidence of HNC. Besides, there should be no direct causal relationship between income and HNC. Instead, the previously observed association between lower income and increased HNC risk should be mediated by education, as those with higher income tend to obtain higher educational levels.

Once diagnosed with HNC, the disease and therapy can impede physical function, result in deformity, and cause psychological suffering (46). Return to work (RTW) is a critical issue for HNC patients since incapacity to RTW is more prevalent in HNC than in other cancer types, and patients with HNC were more likely to quit their employment than patients with other types of cancer (47).

However, by increasing healthcare providers’ awareness of patient factors affecting RTW, such as sociodemographic, psychiatric, and disease-related work aspects, and by designing multidisciplinary interventions, many patients with HNC may return to work (46). Most hospitalization costs for HNC patients are covered by Medicare, alleviating the financial burden on patients (48, 49). After a patient is discharged from the hospital, the cost of therapy gradually decreases, reducing the cost burden on the patient (50). Thus, HNC is unlikely to have had a major impact on patient income due to the effective increase in RTW. This is because the expense of therapy is covered by a national healthcare system. Additionally, income should be mainly determined by education and is usually fixed before catching an HNC.

This study has several strengths: (1) the sample size is relatively large for each GWAS, and the statistical power is sufficient; (2) MR is a robust method of causal inference and can avoid reversing causation. However, some limitations should be pointed out: (1) Horizontal pleiotropy is a natural flaw of MR, and it should affect the results undetectably, though we used various methods to reduce it; (2) we revealed no difference in outcomes between control subject sources, which mitigates the potential for selection bias; (3) another limitation of our analysis was the absence of data from Asia, notably from South East Asia, which has a high rate of head and neck cancer. The most recent study pinpointed that risk factors for oropharyngeal cancer should include tobacco and alcohol consumption, low- and middle-income countries, sex behavior, and HPV infection (3). Another study, which used the latest Global Burden of Disease (GBD) study data, revealed that South Asia had the highest age-standardized incidence rate (ASIR), while East Asia exhibited the highest estimated annual percentage change (EAPC), followed by the high-income Asia Pacific region (4). This study also highlighted that the risk of oropharyngeal cancer is increasing in females, those aged 15 to 49 years, and people from low/middle-income countries. It should be noted that East Asians are likely to be exposed to betel nuts and tobacco, which would deteriorate the prognosis of HPV-positive oropharyngeal cancer (51). Using additional databases could have resulted in the identification of additional publications and a more precise identification of trends (4). The RTW might be a proxy assessment of income since the incapability of RTW caused by oropharyngeal is common; however, the RTW is limited in explaining income as income is usually fixed before diagnosis and the current rate of RTW is increasing (5). The last limitation is that due to the lack of individual-level data, we cannot assess the causal associations of oral sex preference with oropharyngeal cancer, and the HPV infection status for each participant is still unknown. However, we deemed that our MR analysis evaluated the direct causal associations of education and income with the risk of oropharyngeal cancer but did not estimate the indirect causation (22, 52), which might be mediated by HPV infection due to oral sex. Such a mediation analysis needs the GWAS information on oral sex, but unfortunately, it is unavailable. Thus, we cannot rule out the potential indirect causal associations of high income and education with oropharyngeal risk, and they should be investigated soon.

## Conclusion

Our study indicates that either genetically determined educational attainment or household income can affect the risk of oropharyngeal cancer. Furthermore, oropharyngeal cancer does not affect a patient’s degree of education or income. This study highlights that previously reported associations should not be causal.

## Data availability statement

Publicly available datasets were analyzed in this study. This data can be found here: https://www.ebi.ac.uk/gwas/.

## Ethics statement

Ethical review and approval was not required for the study on human participants in accordance with the local legislation and institutional requirements. Written informed consent from the participants was not required to participate in this study in accordance with the national legislation and the institutional requirements.

## Author contributions

WL initiated the idea and designed the whole study. LQ performed the main analyses and wrote the original manuscript. WB checked the whole analytic process and substantially revised the manuscript. SW and XD revised the manuscript and advised on statistical methods. WL was responsible for the accuracy and integrity of this study. All authors contributed to the article and approved the submitted version.
